# Poly(styrene)/oligo(fluorene)-intercalated fluoromica hybrids: synthesis, characterization and self-assembly

**DOI:** 10.3762/bjnano.5.254

**Published:** 2014-12-19

**Authors:** Giuseppe Leone, Francesco Galeotti, William Porzio, Guido Scavia, Luisa Barba, Gianmichele Arrighetti, Giovanni Ricci, Chiara Botta, Umberto Giovanella

**Affiliations:** 1 CNR, Istituto per lo Studio delle Macromolecole (ISMAC), via E. Bassini 15, 20133 Milano, Italy; 2 CNR, Institute of Crystallography, UOS Trieste, Strada Statale 14, 34149 Basovizza, Trieste, Italy

**Keywords:** breath figures, fluoromica, layered silicates, oligo(fluorene), photostability, self-assembly

## Abstract

We report on the intercalation of a cationic fluorescent oligo(fluorene) in between the 2D interlayer region of a fluoromica type silicate. The formation of intercalated structures with different fluorophore contents is observed in powders by synchrotron radiation XRD. Successively, the hybrids are dispersed in poly(styrene) through in situ polymerization. Such a procedure allows us to synthesize the materials from solution, to achieve solid films, and to characterize them by optical and morphologic techniques. The polymeric films with homogeneous distribution of the hybrids exhibit ultraviolet–blue photoluminescence with a significantly enhanced photostability compared to the bare oligo(fluorene)s. Finally, under specific conditions, the polymer hybrid with higher oligo(fluorene) content spontaneously assembles into highly ordered microporous films.

## Introduction

The functionalization of inorganic structures is an effective approach for enriching the potential applications of existing nanomaterials [1–7]. Among the inorganic nano-scaled materials, layered silicates have been widely used as hosts for functional π-conjugated molecules (dyes) [8–10], and polymers [11–15], owing to their adsorption properties, ion-exchange ability, high specific surface area, and a two-dimensional (2D) expandable interlayer space. The combination of these features permits the easy tuning of the interaction between the emitting centers by surface chemistry (i.e., ion-exchange and grafting reactions), and a sandwich-type intercalation. In particular, the intercalation of functional molecular species within the silicate interlayer region is expected (i) to improve the photo-, thermo-, and chemical stability of the dye, which is generally insufficient for a use in applied optoelectronic devices, and (ii) to control the accommodation of the guest for organizing efficient dye assemblies, thus allowing the tuning of the photo-functions of the hybrid [16–17].

**Figure 1:**
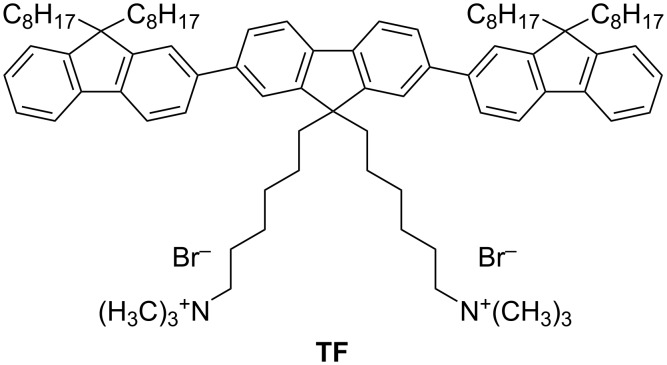
Chemical structure of the cationic oligo(fluorene) (TF).

Herein we report the intercalation of a cationic oligo(fluorene) (Figure 1) in between the interlayer region of a fluoromica type silicate. A series of three samples has been synthesized with different amounts of the dye with respect to the fluoromica maximum cation exchange capacity (CEC), and the formation of intercalated structures has been observed in powders by synchrotron radiation X-ray diffraction (XRD). Successively, in order to enhance the solution processability of the material, the resulting intercalated hybrids were dispersed in a poly(styrene) (PS) matrix by in situ thermal polymerization. Such a procedure allowed us to process the materials as solid films and to characterize them by optical, structural, and morphologic analyses. In addition, we explored the possibility of organizing these materials in ordered honeycomb structures through a self-assembly approach.

## Results and Discussion

### Synthesis and characterization of TF-intercalated SME Hybrids

Sodium-exchanged Somasif ME100 (SME) has been chosen to intercalate a fluorescent oligo(fluorene) cation (hereafter named TF) thanks to its very low iron impurities and low charge density [18–20]. The organo-modified SME hybrids were synthesized by a cation-exchange reaction following the procedure reported elsewhere [8]. In Table 1 the amount of charged TF with respect to the CEC of pristine SME is shown.

**Table 1:** Cation-exchange reaction conditions, and XRD results.

sample	cation exchange	XRD data			

	(% vs CEC)	2θ (°)	*d* spacing^b^ (nm)	*d-*free^c^ (nm)	*L*^d^ (nm)

SME^e^		7.26/9.36	1.22/0.95		50/45
DHS^e^		9.40/9.20	0.94		
T5	5	5.88/7.1	1.51	0.55	10
T15	15	2.65/5.85^e^	3.3/1.51	2.34/0.55	13/30
T30	30	2.87/5.75/8.5^f^	3.15	2.18	13

^a^The XRD data for SME and DHS, reported in [9], are added to help the comparison with the TF-intercalated samples. Peaks other than those indicated observed in the patterns are possibly attributable to a mixed reflection of fluoromica [20]. ^b^The basal spacing, *d*(001), is determined by Bragg’s equation 2*d*(*hkl*) sin θ = λ. ^c^The lamellae clearance (*d-*free) is determined by subtracting the thickness of the SME layer (0.94 nm) from the basal spacing determined from the (001) diffraction peak. ^d^*L* is the crystallite size determined by using the package described in [21]; ^e^Two spacings related to different arrangements are observed. ^f^Three orders of [00*l*] are detected.

### Structural investigation through XRD studies

**Figure 2:**
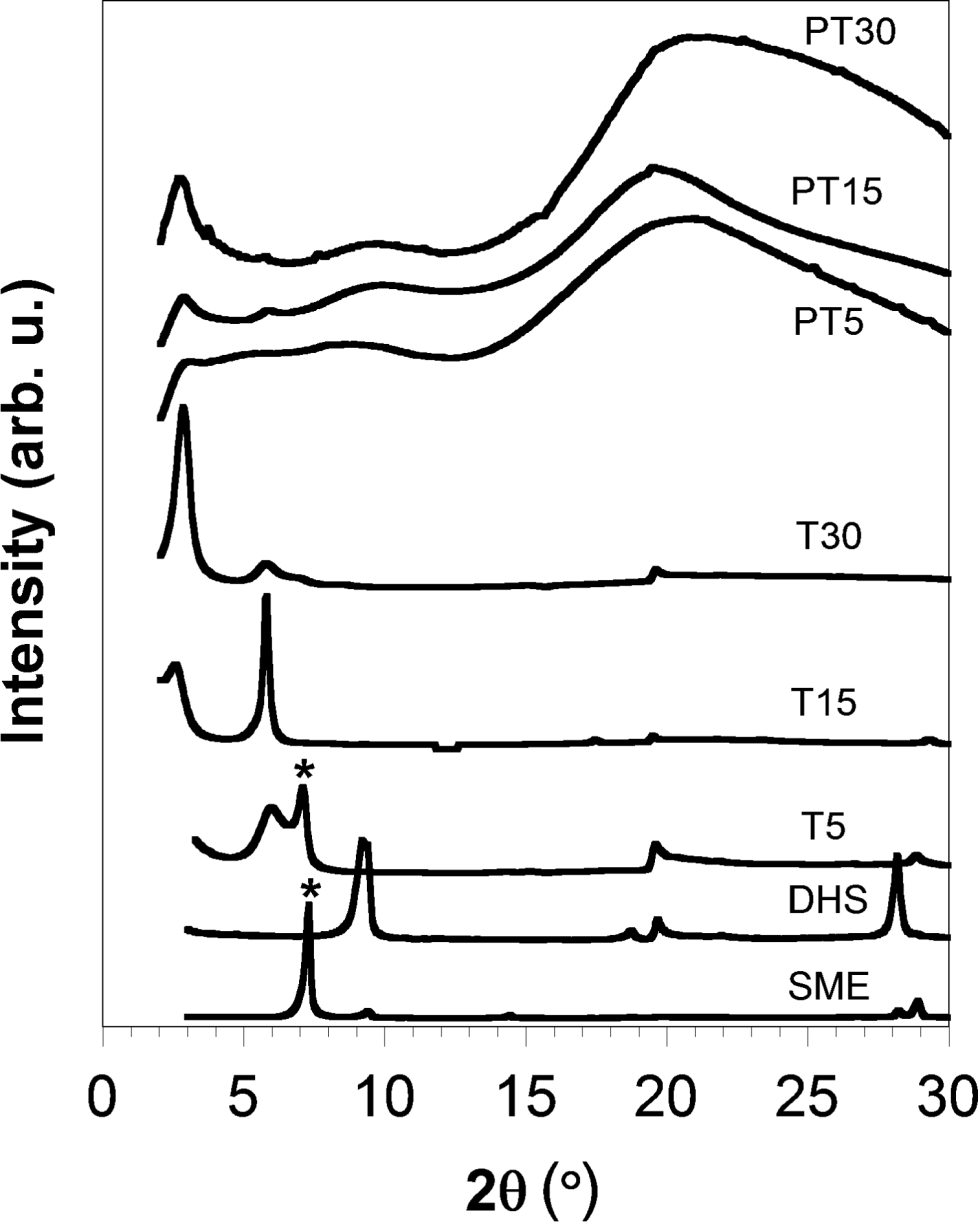
XRD spectra of SME, dehydrated SME (DHS), TF-intercalated hybrids (T5, T15, and T30), and the PS/TF–intercalated SME materials (PT5, PT15 and PT30).

Evidence of the intercalation of the oligo(fluorene) (TF) cation was first provided by XRD analysis (Figure 2 and Table 1). The sample profiles have been treated according to Enzo et al. [21] to derive relevant parameters reported in Table 1. As reported on our previous works [8–9], SME shows two diffraction peaks at 7.30 and 9.40°, corresponding to the interlayer spacing of 1.22 and 0.95 nm of hydrated and dehydrated layers, respectively, while for DHS (dehydrated SME) only the peak at about 9.40° [*d*(001) = 0.95 nm] is observed.

The XRD pattern of TF-intercalated SME samples (T5, T15, and T30) are reported in Figure 2. T5 exhibits a broader peak centered at ca. 5.88°, and a second one at about 7.1°. The former (marked with an asterisk and equivalent to pristine SME [9]) stems from the layer which retains water molecules (meaning that the cation-exchange is incomplete), while the latter reflection, shifted to lower a Bragg angles compared to SME (7.30°), is associated with a layer repetition of 1.46 nm (*d*-free value of 0.51 nm) thus suggesting that the TF cation is intercalated in between the SME galleries. To propose a reasonable interlayer arrangement of TF molecules, the steric limitations between TF and the SME charged sites should be examined. Indeed, steric limitations are determined by the SME equivalent area for the charge deficit layer (*A*_e_) and the minimum area demand (*A*_d_) of intercalated molecules. In the case of sample T5, according to the XRD data (*d*-free = 0.51 nm), and TF size [22], a monolayer arrangement with the alkyl chains extending nearly parallel to the SME lamellae is in agreement with XRD data. In such a configuration the evaluated *A*_d_ value of the oligo(fluorene) can range from 0.4 to 0.5 nm^2^ according to the available space to extend the lateral chains, which is smaller than the SME equivalent area for the charge deficit layer (assuming an average layer charge density of 0.77 nm^2^ as reported by Choy et al. [23]).

When the TF loading is increased up to 15% and 30% CEC the XRD spectra change. T15 shows two uncorrelated peaks at 2.65 and 5.85°, indicating that different intercalating arrangements are obtained (in a forthcoming paper a detailed analysis will be reported). T30 exhibits a diffraction peak having a maximum at 2.9°, strongly shifted to lower angle with respect to the neat SME, which indicates a successful oligo(fluorene) intercalation. An approximate doubling of the interlayer height up to 3.1 nm was observed, and it can be explained if a tilted interlayer distribution of the TF cation is considered. Indeed, such an interlayer spacing (*d*-free = 2.14 nm) is attributable to the intercalation of TF molecules with a position far from flat inside the SME interlayers, and it becomes coherently positioned with second and third order appearance [*d*(002) = 5.7° 2θ, *d*(003) = 8.5° 2θ, see also Table 1]. This fact possibly suggests that a sequence of ordered TF molecules standing-up between layers is formed. For such an inclination, the evaluated *A*_d_ can exceed 1 nm^2^ for each TF molecule; therefore, considering an average layer charge density of 0.77 nm^2^, the area available of SME surface is oversaturated by the oligo(fluorene) cation with a loading of 30% CEC.

### Synthesis and characterization of PS/TF-intercalated SME hybrids

Aiming to improve the processability of the TF-intercalated SME material in solution, we believed that an intriguing strategy would be to use a polymer as dispersing agent. Our previous results [8–10] caused us to regard the in situ polymerization as a potent tool to improve the intimate mixing between the polymer and the inorganic component. Thus, we synthesized the polymer directly by mixing the TF-intercalated SME hybrid with styrene monomer that polymerizes when the temperature is increased to about 120 °C.

The PS/TF-intercalated SME materials (hereafter named PT5, PT15, and PT30) were first characterized by XRD analysis. All the materials still show the presence of a diffraction peak in the low-angle region, clearly related to the precursor features. Similarly to what observed earlier, no XRD peaks of pristine crystalline SME were observed after the polymerization due to the distribution of intercalated SME tactoids within the continuous polymer phase [24–25]. As an example, the XRD pattern of the PT30 material, included in Figure 2, still reveals the presence of a peak in the low-angle region that corresponds to a layer periodicity of 3.14 nm. This peak is only marginally shifted to a lower diffraction angle than that of T30 (*d*_001_ = 3.10 nm), which is consistent with the partial intercalation of PS in between the enlarged SME layers.

### Optical and morphological properties

The emission properties of the polymer hybrids were recorded in 220–230 nm thick spin-coated films. The fluorescence spectra of PT5, PT15, and PT30 films show maxima at 400 nm similar to neat TF, in turn unchanged compared to the terfluorene [26], with vibronic side bands typical of fluorene-based oligomers.

**Figure 3:**
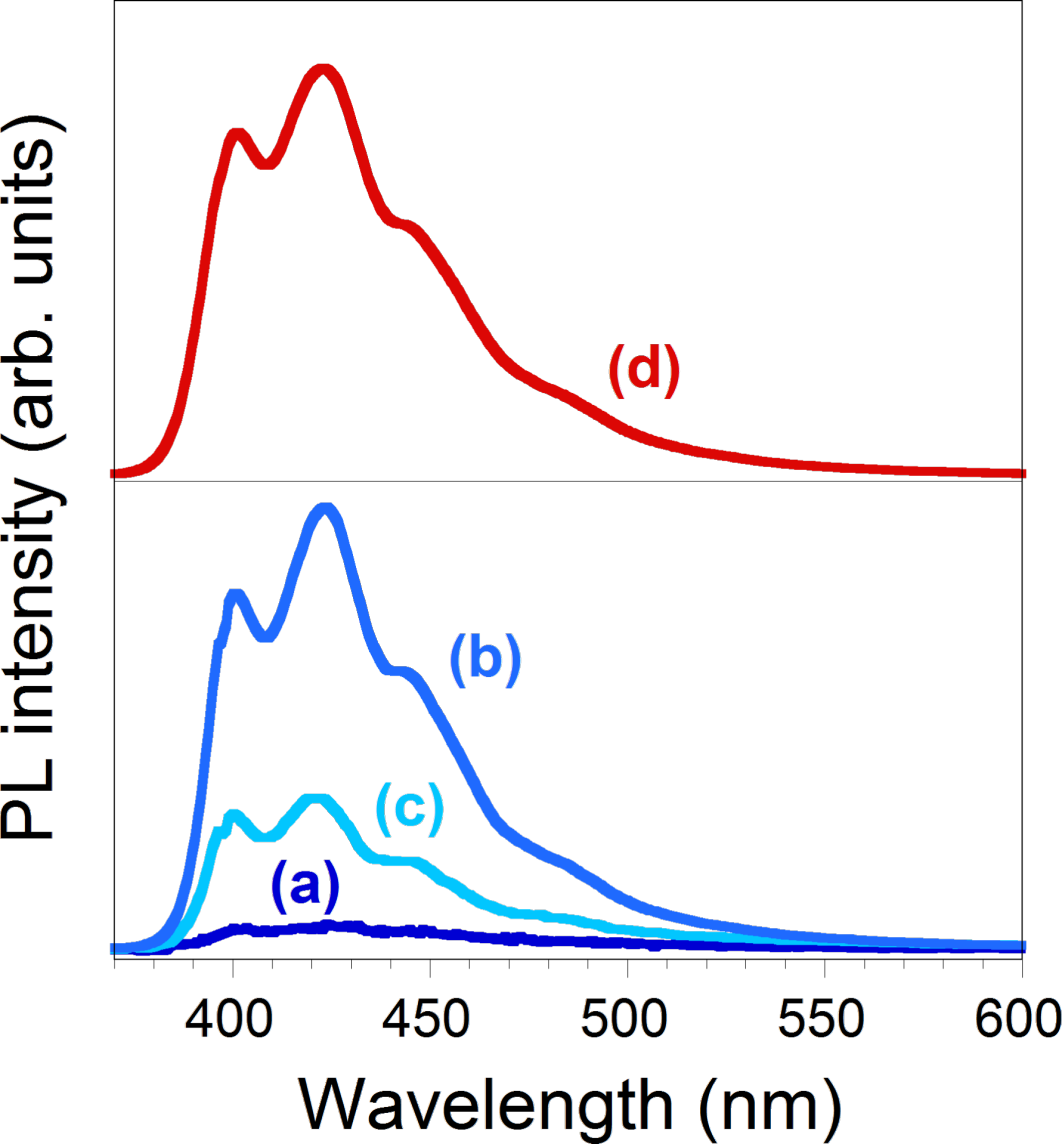
The fluorescence spectra of PT5 (a), PT15 (b), PT30 (c) and TF films (d).

The PL intensity of PT15 film is higher than PT5 and PT30 likely due to the higher density of fluorescent intercalated tactoids. We recorded a PL quantum yield of 48% for PT15 (Figure 3).

**Figure 4:**
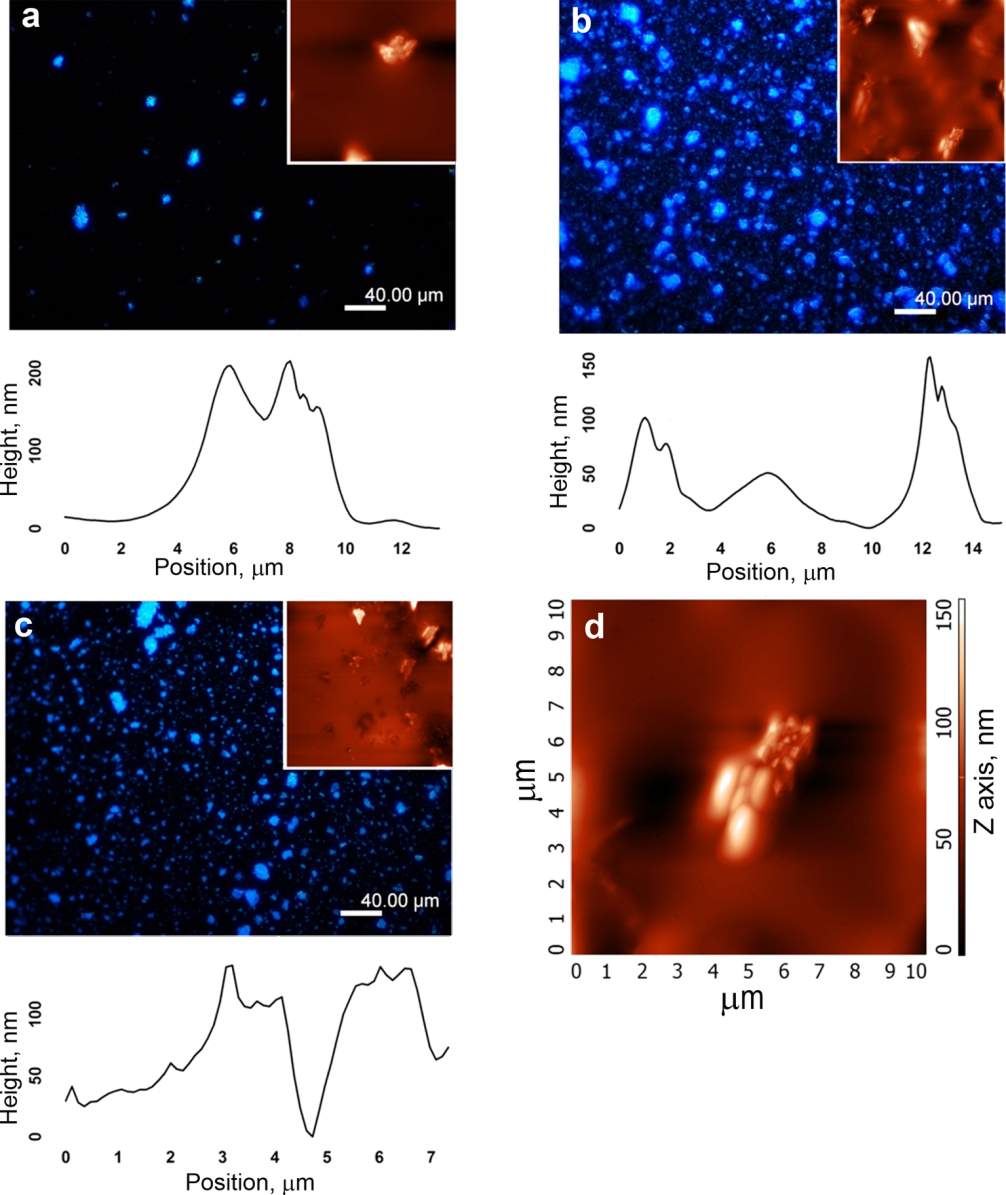
Fluorescence microscopy image, corresponding AFM magnification X,Y = 20 µm (inset) and cluster profile of PT5 (a) PT15 (b) and PT30 (c) films. d) AFM detail of a typical cluster.

Thicker films of PT5, PT15 and PT30 prepared by drop casting of toluene solutions, show a distribution of SME aggregates ranging from a few micrometers to a few tens of micrometers. As evidenced by the fluorescence microscope images in Figure 4, the blue emission comes solely from the silicates, while the polymer matrix is completely dark, which confirms that the whole amount of the emissive TF is confined in between the fluoromica layers. The AFM images reported as inset of Figure 4a–c confirm the large-scale morphology observed by fluorescence microscopy and reveal an organization and orientation of most of the silicates forming clusters (Figure 4d).

### Self-assembly

We explored further possibilities of organization of these materials by drop casting dilute solutions in carbon disulfide under humid atmosphere. Under these conditions, the fast evaporation of the solvent and its consequent cooling trigger the condensation of micrometric water droplets on the polymer solution surface, which leads to the spontaneous formation of breath figure (BF) patterns [27]. This self-assembly technique allows one to create patterned surfaces with highly regular geometry, in an custom-built microfabrication system. Such patterns hold great promise for several up-to-date applications, including nanostructures for optoelectronic devices [28–30], microfiltration membranes [31–32], and plasmonic sensors [33].

**Figure 5:**
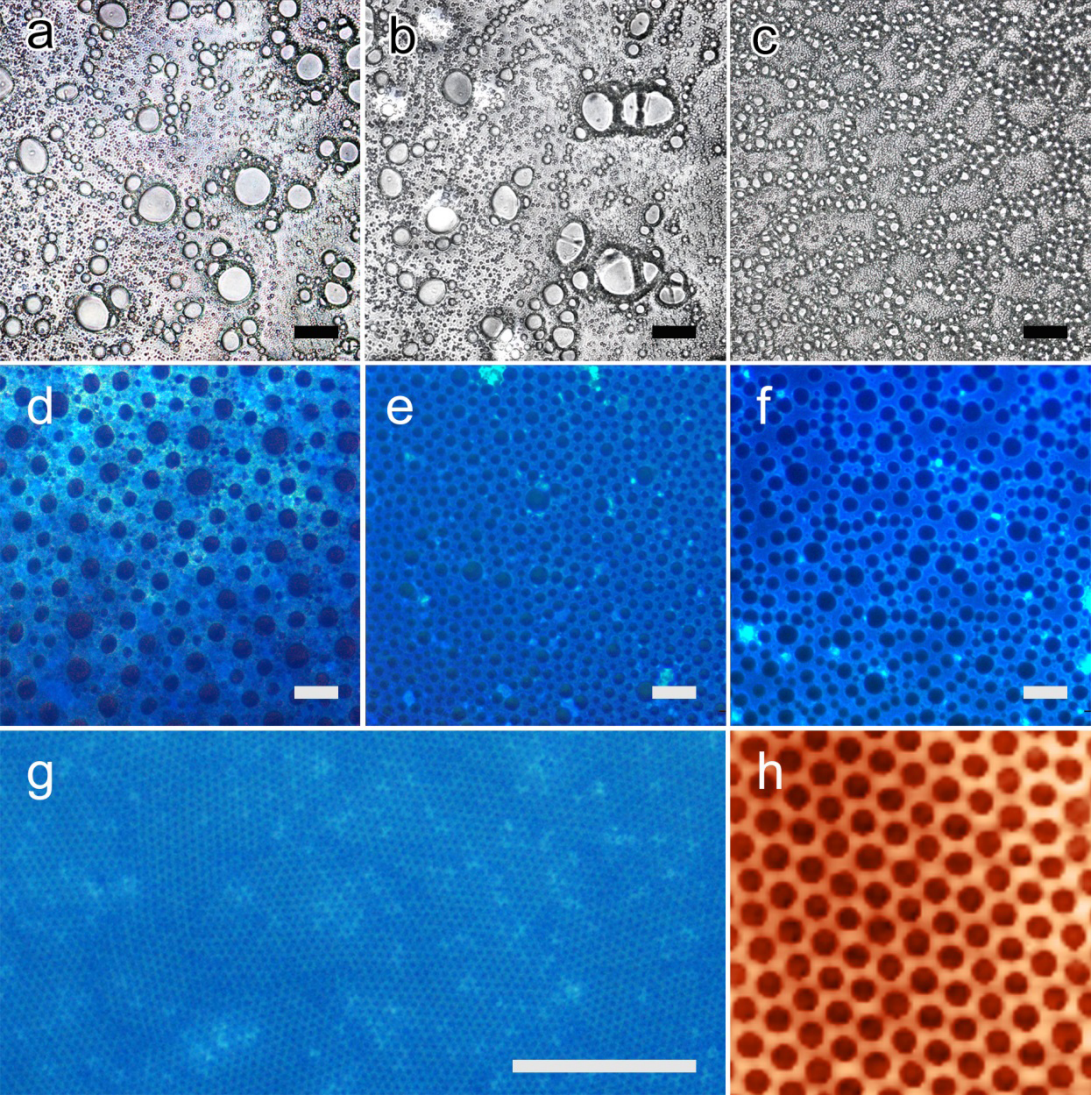
(a–c) Microscopy images of films of PT5, PT15 and PT30 cast under breath figure conditions. (d–f) Fluorescence microscopy images of PT5, PT15 and PT30 films prepared under the same conditions, after the addition of free TF (0.4% w/w). (g) Highly ordered microporous film of PT30 after optimization of parameters. (h) AFM view of a 10 × 10 µm^2^ area of the same film. All scale bars are 20 µm long.

In a recently published study [11], we applied the BF pattering technique to a hybrid copolymer formed by a PS backbone and oligo(fluorene) branches, partially intercalated within the SME layers. The balanced combination of flexible coil, rigid rods, and silicates realized in that single material allowed us to prepare highly ordered BF patterns. By contrast, when we tried to organize PT5, PT15, and PT30 by using the same approach, we could not observe any ordered patterns. As shown in Figure 5a–c, all the films show unpacked and non-ordered cavities, with a wide diameter distribution, resembling what is normally obtained by casting linear PS without polar groups under the same conditions [34]. This is a clear indication that the polymer is not able to stabilize the water droplets forming at the solution/air interface, so that the microdroplets are free to float around and to coalesce in a disordered way. In order to increase the hydrophilicity of the system and hence the ability of the material to stabilize the water droplets, we added some free TF to the polymer solution.

As soon as a minimal amount of free TF is added to the system (0.02 mg·mL^−1^, corresponding to 0.4% w/w with respect to the polymer hybrid), we could observe the formation of the densely packed cavities typical of BFs on the surface of the three films. The micrographs in Figure 5d–f show films of the three materials, which now emit blue light because of the presence of free TF blended with PS, homogeneously covered by cavities of 5–20 µm diameter. Even though the diameter distribution is not uniform in these films, the presence of free TF clearly aided the formation of BF. Underneath, the emitting SME clusters containing intercalated TF are still visible.

The process of BF formation is regulated by different parameters (polymer concentration, cast volume, solvent evaporation rate) that can be tuned to control both the degree of order in the arrangement of the micropores and their size [27]. Figure 5g shows a honeycomb film of PT30 prepared after the optimization of BF parameters; in particular, the concentration of TF blended with PT30 was raised up to 0.2 mg·mL^−1^, and the flow rate of humid nitrogen was set to 400 L·h^−1^. As evidenced by the AFM detail in Figure 5h, in this film cavities have an external diameter of 0.65 µm and a pitch of 1.0 µm and are arranged in a highly ordered hexagonal fashion, while bright SME aggregates are visible under the honeycomb structure, which indicates that a hierarchical organization of this material by the BF approach is feasible.

### Photostability

**Figure 6:**
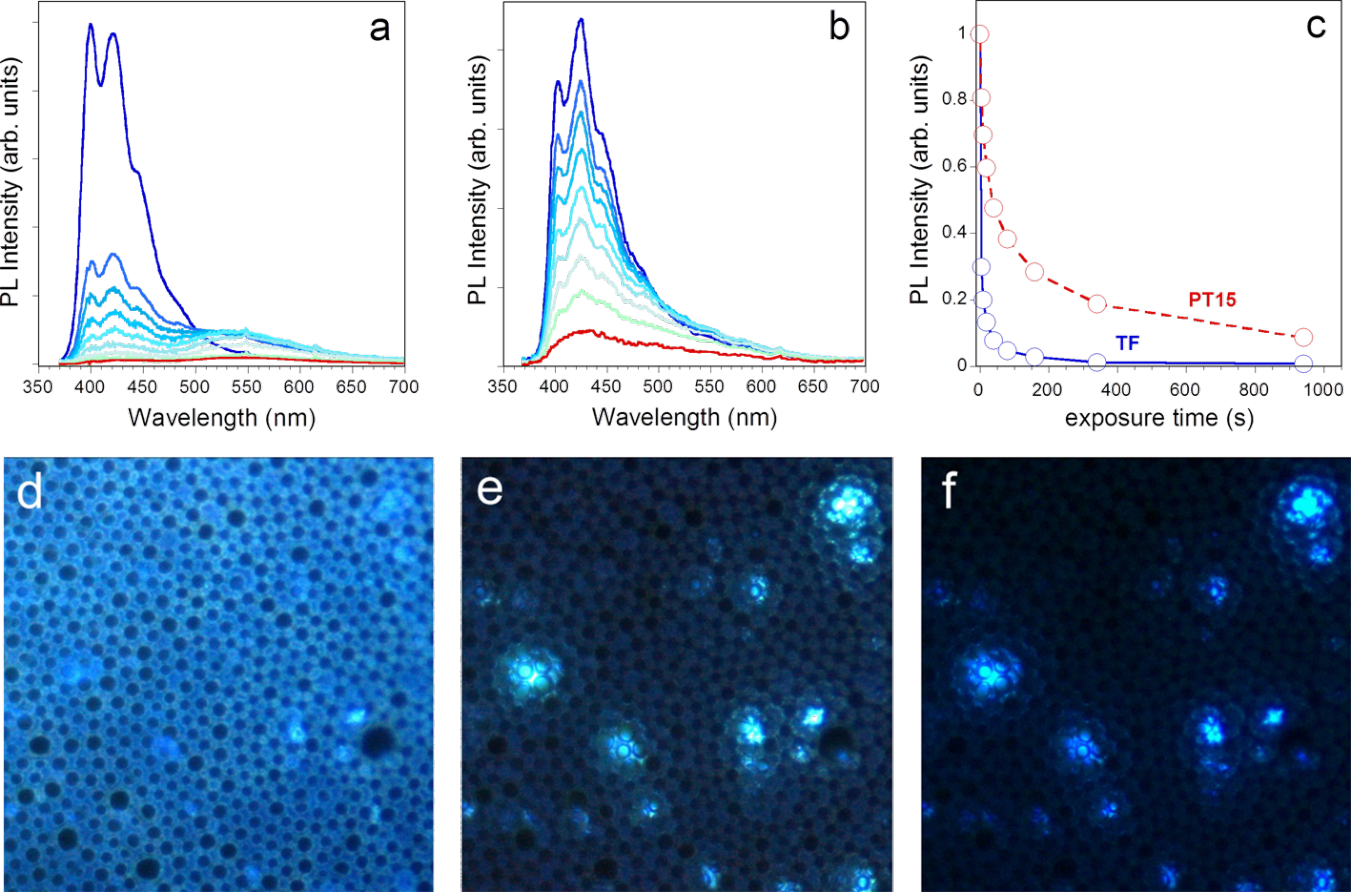
Chromatic stability of steady-state PL spectra upon UV exposure for 0 (dark blue line) to 20 min (red line) of neat flat TF film (a) and PT15 film (b). PL intensity of 400 nm peak of TF and PT15 film versus exposure time to UV light (c). PL images of the patterned PT15 film acquired during exposure to UV light are reported in (d–f).

The intercalation of the oligo(fluorene) molecules within the lamellae interlayers of the inorganic scaffold has dramatically improved their photophysical stability, a critical issue for fluorene-based materials [35]. PT15, selected as a representative sample, shows good chromatic stability when irradiated by a 100 mW/cm^2^ UV lamp at 365 nm (Figure 6b), compared to the flat film of neat TF (Figure 6a). The photodegradation of fluorene-based compounds leads to a reduction of PL intensity together with the appearance of the keto-defects green emission band, peaked at around 530 nm, at the expense of the initial blue emission. In the flat PT15 film the contribution of keto-defects emission is almost suppressed and the decrease of PL intensity is slower (Figure 6c) with respect to neat TF film. In the nanoporous film (Figure 6d–f), the oxidation affects free TF oligomers dispersed in the polymer hybrid, while sharp blue emission from the intercalated TF is still observed, confirming the protecting role played by the silicate.

## Conclusion

In summary, novel inorganic–organic hybrid composites have been prepared by intercalation of a cationic fluorescent oligo(fluorene) in between fluoromica-type silicate layers. The confined arrangement of the emitter is easily tuned by modulating the dye loading as demonstrated by XRD studies. Successively, the hybrids have been in situ dispersed in a PS matrix as a mean of making a composite that is processable from solution.

The hybrid material films exhibit ultraviolet-blue photoluminescence with a significantly enhanced photostability with respect to the bare oligo(fluorene), thanks to the intercalation of the dye in between the inorganic layers which inhibits the photochemical oxidation by blocking the oxygen penetration.

The possibility to organize the polymer nanocomposite by BF technique opens to intriguing applications such as optoelectronic devices, microfiltration membranes, and plasmonic sensors.

## Experimental

### Reagents

Sodium-exchanged Somasif ME100 (SME, CO-OP Chemicals, CEC = 1.2 mmol·g^−1^) was dried at 130 °C under reduced pressure (10^−3^ bar) for 2 weeks and then it was stored under nitrogen. Ethyl alcohol (Carlo Erba, 96% pure) was degassed under vacuum then by bubbling nitrogen, kept over molecular sieves and used without any further purification. Styrene (Aldrich, 99% pure) was refluxed for 4 h over CaH_2_, then distilled trap-to-trap and stored under nitrogen. Distilled deionized water was used for all ion-exchange experiments. 2,7-dibromofluorene, 1,6-dibromohexane and trimethylamine were purchased from Sigma–Aldrich. 9,9-di-*n*-octylfluorene-2-boronic acid pinacol ester was purchased from Alfa Aesar. 2,7-dibromo-9,9-bis(6’-bromohexyl)fluorene was synthesized following the procedure reported elsewhere [36].

### Materials preparation

#### Synthesis of 2,7-bis(9,9-dioctylfluorene-2-yl)-9,9-bis[6-(trimethylammonium)hexyl]fluorene dibromide (TF)

TF was synthesized by standard Suzuki coupling reaction of 2,7-dibromo-9,9-bis(6’-bromohexyl)fluorene and 9,9-di-*n*-octylfluorene-2-boronic acid pinacol ester and subsequent quaternization of the neutral precursor with trimethylamine, according to the following procedure. A mixture of 2,7-dibromo-9,9-bis(6’-bromohexyl)fluorene (173 mg, 0.266 mmol), 9,9-di-*n*-octylfluorene-2-boronic acid pinacol ester (302 mg, 0.585 mmol), Pd(PPh_3_)_4_ (6 mg, 0.005 mmol), aqueous potassium carbonate (2 M, 1.0 mL), and toluene (2.5 mL) was deoxygenated and then heated to 90 °C under nitrogen. The mixture was stirred for 48 h and then cooled to room temperature. The organic fraction was dried over Na_2_SO_4_ and purified by silica gel column chromatography, to afford 300 mg of product as pale powder (yield 87%). Condensed trimethylamine (2.5 mL) was added dropwise to a solution of the neutral precursor polymer (100 mg) in 7.5 mL of THF at −30 °C. The mixture was then allowed to warm up to room temperature for 30 min, and then cooled down again to −30 °C. More trimethylamine (5 mL) was added and the mixture was stirred at room temperature overnight. The obtained transparent gel was dried under a flux of N_2_ to obtain TF as a white solid (93 mg; yield 96%).

1H NMR (600 MHz, CD_3_OD) δ 7.85–7.69 (m, 14H, fluorene ring), 7.38–7.33 (m, 6H, fluorene ring), 3.14 (t, 4H, -C*H_2_*N-), 2.98 (s, 18H,-NC*H_3_*), 2.22–2.08 (m, 12H, C(C*H_2_*-)_2_), 1.55 (m, 4H, -C*H_2_*-CH_2_N-), 1.18–0.63 (m, 72H, -CH_2_C_7_*H_15_* and -CH_2_C_3_*H_6_*C_2_H_4_N-); Anal. calcd for C_89_H_130_Br_2_N_2_: C, 77.02; H, 9.44; Br, 11.52; N, 2.02%; found: C, 77.95; H, 9.91; Br, 11.08; N, 1.93%.

#### Preparation of the intercalated SME hybrids

The synthesis of T30 is reported as standard procedure. To a 100 mL three-neck round bottom flask were added SME (50 mg) and H_2_O (20 mL) and stirred for 5 days at room temperature. Meanwhile, an EtOH solution of TF (17 mg, 1.2 × 10^−5^ mol, 30% vs CEC) is prepared and then added to the SME suspension. The cation-exchange reaction proceeds for 3 h at 60 °C and then for additional 48 h at room temperature. The suspension was filtrated and washed with a H_2_O/EtOH mixture (1:1) to collect the hybrid materials. Once the solvent was removed under reduced pressure, the product was extracted with EtOH by Soxhlet extraction for 8 h. The residual fraction was dried in vacuum and then ground in an agate mortar. T5 was prepared with the loading of 5% of CEC for TF (2.8 mg, 2.0 × 10^−6^ mol) and T15 was prepared with the loading of 15% of CEC for TF (8.1 mg, 5.9 × 10^−6^ mol) for 50 mg of SME.

The preparation of PT30 filled with T30 is reported as a standard procedure. The polymerization experiments were carried out in a 25 mL round-bottomed Schlenk flask, which had been dried on the high vacuum line by heating at 110 °C. The reactor was charged with T30 (10 mg) and styrene (2.20 mL, 1.98 g). The polymerization was carried out at 125 °C for 100 min. When the system was cooled to room temperature, polymerization was stopped by addition of methanol (20-fold excess). The precipitated polymer was collected by filtration, repeatedly washed with fresh MeOH and dried in vacuum to constant weight (yield = 0.615 g; styrene conversion = 31%; *M*_w_ = 30.3 × 10^4^ g/mol; *M*_w_/*M*_n_ = 2.0)

#### Preparation of films

Films for optical characterization were obtained by casting or spin-coating a 20 mg·mL^−1^ toluene solution of the compound on a glass substrate. Honeycomb structured films were obtained by following the procedure reported elsewhere [11], with few optimizations. In particular, to set the optimal conditions for BF formation, concentration of PT5, PT15 and PT30 was varied from 5 to 20 mg·mL^−1^, while free TF was varied from 0.02 to 0.2 mg·mL^−1^.

### Characterization techniques

Size exclusion chromatography (SEC) measurements were carried out on a Waters SECV2000 system equipped with two PLGel Mixed C columns, a 2414 RI detector and a 490 UV diode-array detector. THF was used as solvent and poly(styrene) with molecular weights (*M*_w_) ranging from 162 to 3.28 × 106 g·mol^−1^, as standards. GIWAXS measurements were performed at the X-ray diffraction beamline 5.2 at the synchrotron radiation facility Elettra in Trieste (Italy). The X-ray beam emitted by the wiggler source on the Elettra 2 GeV electron storage ring was monochromatized by a Si(111) double crystal monochromator, focused on the sample and collimated by a double set of slits giving a spot size of 0.2 × 0.2 mm. Both spin-coated films (50–80 nm thick) and powders inserted into a sealed capillary were examined at 25 °C. The beam was monochromatized at energies of 8 keV for films or 10.33 keV for powders. The samples were oriented by means of a four-circle diffractometer with a motorized goniometric head. The X-ray beam direction was fixed, while the sample holder could be rotated about the different diffractometer axes, in order to reach the sample surface alignment in the horizontal plane containing the X-ray beam by means of laser light reflection. Subsequently it was possible to rotate it around an axis perpendicular to this plane or, alternatively, to vary the angle between beam and surface (angle of incidence). Bidimensional diffraction patterns were recorded with a 2M Pilatus silicon pixel X-ray detector (DECTRIS Ltd., Baden, Switzerland) positioned perpendicular to the incident beam, at 200 mm distance from the sample, to record the diffraction patterns in reflection mode. Sample and detector were kept fixed during the measurements. The sample inclination to the beam was changed from ω = −0.05° to ω = 0.25°, in steps of 0.05° yielding seven diffraction images. The *q*-resolution of the 2D images collected was estimated by means of lanthanum hexaboride powder (standard reference material 660a of NIST) and it has been evaluated ranging from 0.2 to 0.3 nm^−1^ both for *q_z_* and *q_xy_*, in agreement with other synchrotron measurements [37–39]. The same calibration standard allowed for the integration of 2D patterns by using the software Fit2D [40] yielding several series of powder-like patterns, corrected for geometry, Lorentz, and beam polarization effects. Peaks positions were extracted by means of the program Winplotr [41]. When sufficient amounts were available, the powders were examined by using an Anton Parr camera under nitrogen flux and a Siemens D-500 diffractometer (Cu Kα radiation, λ = 0.154 nm). The operating voltage and current were 40 kV and 40 mA, respectively. Data were collected from 3 to 33° at 0.05° intervals. PL spectra were recorded by using a Spex 270M monochromator combined with a CCD. UV irradiation of the film was performed by Hamamatsu Lightningcure^TM^ LC8. Atomic force microscopy investigations were performed by using a NT-MDT NTEGRA instrument in semicontact mode in ambient conditions.
